# Hyponatremia in Children With Bacterial Meningitis

**DOI:** 10.3389/fneur.2019.00421

**Published:** 2019-04-30

**Authors:** Feixia Zheng, Xiuyun Ye, Xulai Shi, Zhongdong Lin, Zuqin Yang, Longxiang Jiang

**Affiliations:** ^1^Department of Pediatrics, The Second Affiliated Hospital & Yuying Children's Hospital of Wenzhou Medical University, Wenzhou, China; ^2^Department of Respiratory Medicine, Wenzhou Hospital of Integrated Traditional Chinese and Western Medicine, Wenzhou, China

**Keywords:** hyponatremia, bacterial meningitis, pediatrics, prognosis, neurologic complications, systemic complications

## Abstract

**Background:** Hyponatremia has frequently been described as a common complication associated with bacterial meningitis, though its frequency and clinical course in children with bacterial meningitis are unclear. The present study aimed to investigate the frequency, clinical characteristics, and prognosis associated with pediatric hyponatremia due to bacterial meningitis.

**Methods:** We performed a retrospective review of children with bacterial meningitis provided with standard care. One hundred seventy-five children were included. We documented all participants' symptoms and signs, laboratory and microbiological data, radiological findings, and complications that occurred during their hospital admission. Disease severity was determined using the maximum Pediatric Cerebral Performance Category (PCPC) and minimum Glasgow Coma Scale (GCS). Residual deficits were assessed using PCPC at discharge.

**Results:** Hyponatremia (<135 mmol/L) was seen in 116 (66.4%) of the patients assessed and was classified as mild (130–135 mmol/L) in 77, moderate (125–129 mmol/L) in 26, and severe (<125 mmol/L) in 13. Hyponatremia was associated with a shorter duration of symptoms before admission, higher CSF white cell counts, and a longer duration of hospitalization. Moderate and severe hyponatremia were associated with an increase in convulsions, impaired consciousness, altered CSF protein levels, higher maximum PCPC scores, and lower minimum GCS scores. Severe hyponatremia was further associated with the development of systemic complications including shock, multiple organ dysfunction syndrome, respiratory failure requiring mechanical ventilation, and an increase in poor outcome (PCPC ≥ 2). Hyponatremia was not associated with the development of neurologic complications. Logistic regression analyses revealed that convulsions (OR 12.09, 95% CI 2.63–56.84) and blood glucose levels > 6.1 mmol/L (OR 8.28, 95% CI 1.65–41.60) predicted severe hyponatremia.

**Conclusion:** Hyponatremia occurred in 66.4% of the assessed pediatric bacterial meningitis patients. Moderate and severe hyponatremia affected the severity of pediatric bacterial meningitis. Only severe hyponatremia affected the short-term prognosis of patients with pediatric bacterial meningitis. We recommend that patients with pediatric bacterial meningitis who exhibit convulsions and increased blood glucose levels should be checked for severe hyponatremia. Further studies are needed to evaluate the effectiveness of treatment of hyponatremia.

## Introduction

Hyponatremia, defined as a serum sodium concentration of <135 mmol/L, is particularly common in neurological patients because of the major role of the central nervous system (CNS) in the regulation of sodium and water homeostasis (1). Among disorders of the CNS, hyponatremia is commonly noted in encephalitis, meningitis, subarachnoid hemorrhage, and head injury. However, the incidence of hyponatremia varies vastly among these neurologic diseases (2, 3). Specifically, hyponatremia in the context of infection reflects the severity of the underlying disease state and is associated with prolonged hospitalization and significant morbidity (4).

Bacterial meningitis is a severe infectious disease of the membranes lining the brain, which results in high rates of mortality and morbidity worldwide. Neurologic and systemic complications occur in large proportions of children and adults with bacterial meningitis (5). In particular, hyponatremia during CNS infections may be overlooked by clinicians because cerebral edema also explains the majority of clinical hyponatremia signs and symptoms, which are predominantly neurological.

In the past several decades, the epidemiology of and use of treatment strategies for bacterial meningitis have changed significantly (5). There is a paucity of studies regarding the frequency and clinical significance of hyponatremia in children with bacterial meningitis. Thus, the aim of the present study was to report the frequency, clinical characteristics, and short-term prognosis associated with hyponatremia in patients with childhood bacterial meningitis.

## Participants and Methods

### Study Population

A retrospective review was conducted on the medical records of children <16 years of age who were admitted with bacterial meningitis to the Department of Pediatrics of the Second Affiliated Hospital & Yuying Children's Hospital of Wenzhou Medical University, and who received standard care between December 1, 2015 and March 1, 2018. The study was approved by our ethics committee (IRB approval number: KYKT2018109). Informed consent was obtained from the parent(s) or legal guardian(s) of each patient before initiating medical record review.

Diagnoses of bacterial meningitis were based on the isolation of a specific organism from positive cerebrospinal fluid (CSF) culture or CSF gram stain. In addition, CSF pleocytosis of mainly polymorphic leukocytes, low glucose concentration, low CSF to blood glucose ratio, and elevated protein levels was regarded as bacterial meningitis (5). Patients with primary renal, hepatic, or cardiac failure; pulmonary disease; hypoxic-ischemic encephalopathy; bilirubin encephalopathy; traumatic brain injury; or intracranial hemorrhage in the past month, all conditions which could result in hyponatremia, were excluded from the present study. However, if bacterial meningitis patients developed intracranial hemorrhage or hepatic or renal function derangement during treatment, they were not excluded.

Medications/infusions were mixed in 10% dextrose in water (D10W) or normal saline (NS). Hypotonic saline including D10W-one-fifth NS, D10W-one-third NS, and total/peripheral parenteral nutrition were used as a maintenance fluid when necessary. IV fluids used to correct serum sodium levels included hypotonic saline (D10W-one-half NS), isotonic saline, and hypertonic saline.

### Investigations

Symptoms and signs, patient history, laboratory and microbiological data, radiological examination results, inpatient complications, treatments including sodium-altering therapies, length of stay, and indications for treatment (e.g., hyponatremia and worsening results of neurologic exam) were recorded. Serum sodium concentrations were measured on admission and then again after ≥7 days in all children. In children who had a serum sodium concentration of ≤129 mmol/L, serum sodium concentrations were measured again after ~6 and 12 h, then were measured daily until concentrations stabilized with treatment.

### Hyponatremia

Hyponatremia was diagnosed when sodium concentration fell below 135 mmol/L. The severity of hyponatremia was further defined as follows: mild, 130–135 mmol/L; moderate, 125–129 mmol/L; and severe, <125 mmol/L (1). The lowest recorded level of serum sodium was used to define the severity of each patient's hyponatremia.

### Evaluation

Disease severity was determined using maximum Pediatric Cerebral Performance Category (PCPC) and minimum Glasgow Coma Scale (GCS) values. Patients' outcomes were defined using a neurological examination conducted at discharge. Outcomes were further graded using PCPC. Patients were considered to have a poor outcome if their PCPC score was ≥2 and a good outcome if their PCPC score was = 1. Data were collected until patients were discharged.

### Statistical Analyses

All statistical analyses were conducted using SPSS (version 25, IBM Corp., Armonk, NY, USA). Categorical variables were evaluated using either the chi-squared test or Fisher's exact test. Continuous data were determined to have a normal distribution if the Shapiro–Wilk test had a significance value of >0.05. Continuous variables were summarized using means and standard deviations or medians and interquartile ranges, as appropriate. Non-normally distributed data were evaluated using the Kruskal–Wallis *H* test or Mann–Whitney *U*-test. Normal continuous data were evaluated using ANOVA. Correlations were measured via Spearman's rank testing. Factors predictive of severe hyponatremia were assessed via univariable binary logistic regression, independently of the treatments administered. Variables with a two-tailed *p* < 0.05 were considered to be significant.

## Results

### Demographics

In total, 175 children [94 boys (54%) and 81 girls (46%)] aged from 1 day to 15 years [0.08 (0.05, 0.33) years], of whom 151 (86.3%) were <6 months of age, were studied. A full description of patient demographics is included in Table 1. A total of 118 episodes of hyponatremia were observed in 116 (66.4%) patients, including 99 episodes on admission, and 19 episodes that developed during inpatient treatment; these occurred in similar proportions in all patients with severe, moderate, or mild hyponatremia (*p* = 0.733). Hyponatremia severity varied. Seventy-seven patients (44%) represented the majority of the population classified as mild. In 26 patients (15%), hyponatremia was moderate, whereas it was severe in 13 (7.4%). The severity of hyponatremia across different age groups was similar (*p* = 0.568). No statistically significant differences were observed in the severity of hyponatremia between males and females, although the proportion of male patients with moderate/severe hyponatremia was slightly higher than that in females (27.7 vs. 16%, *p* = 0.066).

**Table 1:** Characteristics of patients with normonatremia and those with mild, moderate, and severe hyponatremia.

**Patient characteristic**		**Normonatremia**	**Mild hyponatremia**	**Moderate hyponatremia**	**Severe hyponatremia**	**Total**
***n***		**59**	**77**	**26**	**13**	**175**
Age	<28 d	22 (31.4%)	31 (44.3%)	10 (14.3%)	7 (10%)	70
	28 d~6 m	27 (33.3%)	38 (46.9%)	13 (16%)	3 (3.7%)	81
	>6 m	10 (41.7%)	8 (33.3%)	3 (12.5%)	3 (12.5%)	24
Sex	Male	33 (35.1%)	35 (37.2%)	18 (19.1%)	8 (8.5%)	94
	Female	26 (32.1%)	42 (51.9%)	8 (9.9%)	5 (6.2%)	81
Etiological organisms	*E. coli*	7 (43.8%)	5 (31.3%)	1 (6.3%)	3 (18.8%)	16
	*S. pneumoniae*	3 (37.5%)	3 (37.5%)	0 (0%)	2 (25%)	8
	*S. agalactiae*	1 (5.3%)	9 (47.4%)	6 (31.6%)	3 (15.8%)	19
	Others	4 (25%)	8 (50%)	2 (12.5%)	2 (12.5%)	16
Sodium concentration in serum (mmol/L)		137.2 (136, 138.3)	133.1 (131.9, 134)	128.15 (126.83, 129)	121.3 (116, 124.05)	

*S. pneumonia, Streptococcus pneumoniae; S. agalactiae, Streptococcus agalactiae; E. coli, Escherichia coli*.

### Etiology

**Figure 1:**
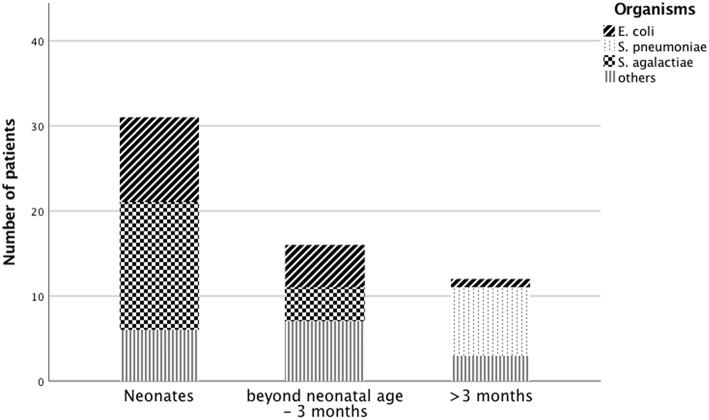
Causative organisms for pediatric meningitis across age groups.

An etiological diagnosis was possible in 59 cases (34%); *Streptococcus agalactiae* had the highest detection rate (Table 1, Figure 1). *S. agalactiae* (48.4%) and *Escherichia coli* (32.3%) were the most common causative pathogens in neonates. *Streptococcus pneumoniae* was the most common causative pathogen in children >3 months of age.

The prevalence of hyponatremia varied based on the infecting organism (*p* = 0.036); it affected 56.3% of patients with *E. coli* meningitis, 62.5% of patients with *S. pneumoniae* meningitis, 94.7% of patients with *S. agalactiae* meningitis (proportion compared with all other patients with organisms detected, *p* = 0.033), and 75% of patients with meningitis caused by other microorganisms (e.g., *Enterobacter cloacae, Klebsiella pneumoniae, Enterococcus faecalis, Flavobacterium meningosepticum, Staphylococcus sciuri, Enterococcus faecium, Enterococcus avium, Streptococcus alactolyticus, Streptococcus gallolyticus* subsp. *pasteurianus*, and *Pseudomonas aeruginosa*).

The proportion of patients with moderate/severe hyponatremia and *S. agalactiae* meningitis was slightly higher than that for meningitis caused by other organisms, although no statistically significant differences were observed (47.4 vs. 25%; *p* = 0.086). Median Na^+^ values during the hospital stay of patients with *S. agalactiae* infection were lower than those for patients with other types of pathogen infections [130.70 (126.30, 133.70) vs. 133.10 (128.95, 137.18); *p* = 0.049]. The prevalences of hyponatremia in neonates with *S. agalactiae* meningitis (93.3% neonatal vs. 100% older; *p* = 1.000) or *E. coli* meningitis (40% vs. 83.3%; *p* = 0.145) were similar to that in children beyond neonatal age.

### Clinical Profiles

The clinical profiles of the children in this study are presented in Table 2. Comorbidities included fever (97.1%), impaired consciousness (56%), anorexia (53.1%), convulsions (21.1%), and vomiting (24%). Patient age, fever status, anorexia, and vomiting were found in similar proportions in patients with severe, moderate, mild, or no hyponatremia, even after excluding patients who developed hyponatremia in the hospital (*p* = 0.081, *p* = 0.591, *p* = 0.405). Moderate and severe hyponatremia were associated with significantly higher frequencies of convulsions (*p* = 0.000) or impaired consciousness (GCS < 14) (*p* = 0.000). Additionally, patients with moderate and severe hyponatremia had lower minimum GCS scores (*p* = 0.000) and higher maximum PCPC scores (*p* = 0.000) than did patients with normonatremia during their inpatient stay. Minimum GCS scores (*r*_*s*_ = 0.34, *p* = 0.000) and maximum PCPC scores (*r*_*s*_ = −0.35, *p* = 0.000) were significantly correlated with serum sodium levels.

**Table 2:** Comparison of characteristics of bacterial meningitis patients with normonatremia and with mild, moderate, and severe hyponatremia.

**Patient characteristic**	**Normonatremia**	**Mild hyponatremia**	**Moderate hyponatremia**	**Severe hyponatremia**	***p*-value**
Age (years)	0.17 (0.04,0.42)	0.08 (0.05, 0.25)	0.08 (0.52, 0.27)	0.07 (0.05, 0.5)	0.944
Anorexia (n)	30/59 (50.8%)	36/77 (46.8%)	18/26 (69.2%)	9/13 (69.2%)	0.142
Vomiting (n)	15/59 (25.4%)	15/77 (19.5%)	9/26 (34.6%)	3/13 (23.1%)	0.462
Fever (n)	56/59 (94.9%)	76/77 (98.7%)	26/26 (100%)	12/13 (92.3%)	0.225
Convulsions (n)	6/59 (10.2%)	14/77 (18.2%)	8/26 (30.8%)	9/13 (69.2%)	0.000
Impaired consciousness (GCS < 14, n)	24/59 (40.7%)	40/77 (51.9%)	22/26 (84.6%)	12/13 (92.3%)	0.000
GCS minimum score	15 (14, 15)	14 (14, 15)	14 (13, 14)	11 (8.5, 13.5)	0.000
PCPC maximum score	1 (1, 2)	2 (1, 2)	2 (2, 2)	3 (2, 4)	0.000
Duration of symptoms prior to admission (days)	3 (2, 4)	2 (1, 3)	2 (1, 4)	2 (1, 3)	0.004
	3 (2, 4)	2 (1, 3)			0.000
Duration of symptom after admission (days)	1.5 (0.5–2.5)	2 (1, 4)	2.75 (1, 5)	5 (3, 5.5)	0.000
	1.5 (0.5–2.5)	2.5 (1, 5)			0.000
Hospital stay (days)	20 (16, 23)	21.5 (18, 36)	21 (15.5, 41.5)	26 (20, 51)	0.044
**LABORATORY TESTS**					
Potassium (mmol/L)	4.85 ± 0.63	4.72 ± 0.59	4.44 ± 0.51	4.19 ± 0.55	0.001
Creatinine (μmol/L)	24.8 (20.6,28.9)	23.9 (21.33,28.15)	28 (22.95,32)	27.25 (22.15,42.53)	0.158
Blood urea nitrogen (mmol/L)	3.16 (2.32, 4.04)	3.56 (2.85, 4.49)	3.08 (2.66, 4.76)	2.62 (2.08, 5.40)	0.125
Urea/creatine ratio	0.12 (0.09, 0.17)	0.14 (0.11, 0.18)	0.13 (0.09, 0.17)	0.11 (0.07, 0.15)	0.088
Uric acid (μmol/L)	182 (134.25, 254)	171 (133,217)	211 (184.75, 224)	128 (79, 189.75)	0.163
Glucose (mmol/L)	5.44 (4.72, 6.33)	5.8 (5.15, 6.91)	6.5 (5.55, 8.25)	7.45 (5.99, 11.83)	0.020
Elevated transaminases (n)	8/59 (13.6%)	11/77 (14.3%)	5/26 (19.2%)	5/13 (38.5%)	0.169
Indexes of CSF					
White cell count (/10^9^l)	164 (68, 660)	550 (170, 1325)	360 (63.75, 4910)	540 (62.5, 3050)	0.068
Protein (g/L)					
>3	6 (10.2%)	10 (13%)	10 (41.7%)	6 (46.2%)	0.000
<3	53 (89.8%)	67 (87%)	14 (58.3%)	7 (53.8%)	
Median (SD) protein (<3)	0.89 (0.54, 1.6)	1.01 (0.6, 1.84)	0.71 (0.55,0.88)	0.92 (0.66,1.52)	0.221
**CLINICAL COMPLICATIONS**					
Shock (n)	1/59 (1.7%)	1/77 (1.3%)	3/26 (11.5%)	3/13 (23.1%)	0.002
MODS (n)	1/59 (1.7%)	0/77 (0.0%)	0/26 (0.0%)	2/13 (15.4%)	0.016
Respiratory failure requiring mechanical ventilation (n)	1/59 (1.7%)	2/77 (2.6%)	0/26 (0%)	3/13 (23.1%)	0.013
Hydrocephalus (n)	4/58 (6.9%)	3/76 (%)	0/25 (0%)	0/12 (0%)	0.630
Subdural fluid accumulation (n)	7/58 (12.1%)	10/76 (13.2%)	1/25 (4%)	2/12 (16.7%)	0.580
Subdural empyema (n)	1/58 (1.7%)	2/76 (2.6%)	0/25 (0%)	1/12 (8.3%)	0.465
Brain parenchymal abnormalities (n)	5/58 (8.6%)	9/76 (11.8%)	2/25 (8%)	1/12 (8.3%)	0.912
**OUTCOMES**					
PCPC score on discharge	1 (1,1)	1 (1,1)	1 (1,1)	1 (1,2)	0.001
Poor outcome (PCPC ≥ 2)	1/59 (1.7%)	2/77 (2.6%)	0/26 (0%)	3/13 (23.1%)	0.013

*^#^Serum potassium levels were measured in 168 patients, creatinine and urea nitrogen in 168 patients, glucose in 89 patients, uric acid in 119 patients, and CSF protein in 173 patients*.

The duration of symptom presentation prior to admission varied from 0.15 to 10 days. The duration of symptom presentation prior to admission in patients with hyponatremia was shorter than in patients with normonatremia [2 (1,3) vs. 3 (2,4) days; *p* = 0.000]. Symptom duration after admission in patients with hyponatremia was longer than in patients with normonatremia [2.5 (1, 5) vs. 1.5 (0.5, 2.5) days; *p* = 0.000]. The duration of hospitalization significantly differed among patients with severe, moderate, mild, and no hyponatremia (*p* = 0.044). Furthermore, there was a significant correlation between the duration of hospitalization and serum sodium levels (*r*_*s*_ = −0.30, *p* = 0.000).

### Laboratory Findings

Serum creatinine levels, urea nitrogen (BUN) levels, uric acid levels, urea/creatine ratios, and the proportions of elevated transaminases were similar in patients with severe, moderate, mild, and no hyponatremia. Patients with moderate and severe hyponatremia had higher serum potassium levels than did patients with mild hyponatremia and normonatremia (*p* = 0.001). Patients with severe hyponatremia had higher glucose levels than did patients with normonatremia (*p* = 0.020). Lumbar punctures were performed in all patients. Furthermore, patients with hyponatremia had a higher CSF white cell count (*p* = 0.008) and those with moderate and severe hyponatremia had elevated CSF protein levels (*p* = 0.000).

### Complications

Severe hyponatremia was associated with a significantly higher frequency of shock (*p* = 0.002), multiple organ dysfunction syndrome (MODS, *p* = 0.016), and acute respiratory failure requiring invasive mechanical ventilation (*p* = 0.013) than normonatremia.

Brain MRIs were performed at least once in 168 patients. In 17 patients, this brain imaging revealed parenchymal abnormalities, including in the periventricular space in six patients, in the frontal lobes in five patients, in the parietal lobes in four patients, in the basal ganglia in two patients, and in the semi-oval center, the splenium of the corpus callosum, the temporal lobes, and the occipital lobes in one patient. The proportions of patients with severe, moderate, mild, and no hyponatremia were similar among those with hydrocephalus, subdural fluid accumulation, subdural empyema, and brain parenchymal abnormalities on MRI. Osmotic demyelination syndrome did not occur in any patient.

### Outcome

Severe hyponatremia was associated with significantly higher PCPC scores on discharge than moderate, mild, and no hyponatremia (*p* = 0.001). A total of 169 (96.6%) patients with BM had good outcomes (PCPC = 1), while six (3.4%) had poor outcomes (PCPC ≥ 2), including four patients who withdrew from treatment due to severe disease conditions. No children died during hospitalization.

The proportions of patients were poor outcomes was similar among patients with *S. agalactiae, S. pneumoniae*, and *E. coli* meningitis (15.8%, 12.5%, 6.3%; *p* = 0.829). Patients with severe hyponatremia had a higher frequency of poor outcomes when compared to those with normonatremia and mild and moderate hyponatremia patients (*p* = 0.013). Multivariate analyses revealed that convulsions (OR 12.09, 95% CI 2.63–56.84, *p* = 0.001) and blood glucose levels >6.1 mmol/L (OR 8.28, 95% CI 1.65–41.60, *p* = 0.01) were related to severe hyponatremia.

## Discussion

The present study reveals that hyponatremia occurred in 66.4% of pediatric bacterial meningitis patients in the present study, the majority of whom were categorized as mild. Hyponatremia was associated with a shorter symptom duration prior to admission, higher CSF white cell counts, a longer duration of symptoms after admission, and a longer hospitalization duration. As expected, moderate and severe hyponatremia were associated with the increased occurrence of some symptoms (conscious disturbance and convulsion), lower minimum GCS scores, higher maximum PCPC scores, and higher levels of CSF proteins, which are clinical indicators of a more severe brain injury and greater degree of pro-inflammation. Severe hyponatremia was also associated with an increase in systemic complications (shock, MODS, and respiratory failure) and poor short-term outcomes. Convulsions and increased levels of blood glucose successfully predicted severe hyponatremia in this cohort of patients.

Very few studies have addressed the frequency, effects, or treatment of hyponatremia in bacterial meningitis. The present study revealed that the most common causative pathogens responsible for this condition in neonates are *S. agalactiae* and *E. coli*, similar to those reported elsewhere (5). Furthermore, the most common causative pathogen in children >3 months of age was *S. pneumoniae* and not *Meningococcus*, which differs from recently reported results (5); this may be because meningococcal vaccines have been used in national routine immunization programs in China, thereby effectively reducing invasive meningococcal disease (6). In the current study, the prevalence of hyponatremia in neonates with *S. agalactiae* meningitis or *E. coli* meningitis was similar to that in children beyond the neonatal period. Previously reported incidences of hyponatremia (serum sodium levels <135 mmol/L) in adults with bacterial meningitis have ranged between 30.3 and 58% at the time of hospital admission (2, 7, 8), while the incidence in children was previously reported to be 50% (10/20) (9). In this study, we found an exceptionally high rate of hyponatremia in children with *S. agalactiae* meningitis (94.7%); notably, a comparably high rate of hyponatremia has also been described in adult patients with *Listeria monocytogenes* (73%) meningitis (7).

Hyponatremia exerts most of its clinical effects on the brain. As the lower extracellular osmolality promotes movement of water into cells, severe, and sometimes fatal, cerebral edema may occur. This happens more frequently when hyponatremia develops in <48 h (1, 4). Experimental studies suggest that the brain needs 48 h to adapt to a hypotonic environment, achieved mainly by extruding sodium, chloride, potassium, and organic osmoles from its cells (1, 10). The adaptation that permits survival in patients with severe, chronic (>48 h) hyponatremia can also cause damage within the brain if the serum sodium concentration is corrected too rapidly. Breakdown of the myelin sheath insulating individual neurons can result in osmotic demyelination syndrome (also known as central pontine myelinolysis and extrapontine myelinolysis), which is a rare but dramatic complication in chronic hyponatremia with rapid correction (1, 10, 11).

Previous reports have indicated that hyponatremia is an independent predictor of increased morbidity and mortality in children with bacterial meningitis (12). In a retrospective study, although hyponatremia in children with bacterial meningitis did not independently predict mortality, it was associated with an increased risk of mortality (13). Moderate and severe hyponatremia (serum sodium levels <130 mmol/L) were associated with an increased risk of mortality in children with pneumococcal meningitis in another retrospective study (14).

Critically, the coexistence of hyponatremia and syndrome of inappropriate antidiuretic hormone secretion (SIADH) in children with bacterial meningitis has previously been associated with higher mortality rates (15). In this study, we found that only severe hyponatremia (serum sodium levels <125 mmol/L) was associated with poor short-term outcomes in children with bacterial meningitis, a result which agrees with those reported previously, also in children (12–15). However, in a nationwide observational cohort study based in the Netherlands, hyponatremia was a common and benign complication in adults with bacterial meningitis. Furthermore, no associations were found with either the severity of disease or outcomes, although an association was found with the duration of symptoms (7). This result differs from those of other pediatric reports. The present study is the first to report that moderate and severe hyponatremia affected the severity of pediatric bacterial meningitis; hyponatremia was associated with the duration of hospitalization in pediatric patients with bacterial meningitis.

Hypokalemia can cause neurological complications in hyponatremic patients via osmotic demyelination syndrome (16, 17). In the present study, patients with moderate and severe hyponatremia showed reduced serum potassium levels, but none developed osmotic demyelination syndrome. Thus, hypokalemia may not commonly contribute to this outcome in patients with pediatric bacterial meningitis who exhibit severe hyponatremia.

The etiological mechanism by which hyponatremia results from bacterial meningitis remains unclear. However, SIADH, cerebral salt wasting syndrome, early or late pituitary dysfunction, and aggressive hypotonic fluid administration are the most probable mechanisms (4, 18, 19). Differentiation of cerebral salt wasting syndrome from SIADH can be difficult. Evidence of fluid volume depletion is critical to making this determination (20, 21). Unfortunately, the assessment of fluid volume status by history and physical examination in children with hyponatremia is often inaccurate.

In addition to those etiologies discussed above, hyponatremia may be caused by osmotic agents, diuretics, and antiepileptic medication toxicity (22, 23). Volume depletion due to nutritional deficiencies, recurrent vomiting, diarrhea, increased insensible water loss, or hyperhidrosis may also be implicated in the pathogenesis of hyponatremia. However, in the present study, hyponatremia on admission was not accompanied by a higher proportion of fever, anorexia, or vomiting, nor was it associated with an extended period of symptomatology prior to admission.

Notably, the administration of hypotonic intravenous solutions may contribute to the development of hyponatremia. For the past 60 years, the prescription for maintenance intravenous fluids for infants and children has consisted of a hypotonic fluid (24, 25). However, in the past 15 years, a multitude of clinical studies have revealed increased risks of hospital-acquired hyponatremia and hospital-aggravated hyponatremia when children receive hypotonic intravenous fluids, compared to isotonic intravenous fluids (25–27). Thus, in 2018, the American Academy of Pediatrics recommended that patients aged 28 days to 18 years requiring maintenance intravenous fluids should receive isotonic solutions with appropriate potassium chloride and dextrose to decrease the risk of hyponatremia (25). The data collected in this study were generated during medical treatment that was performed prior to the publication of this guideline, so hypotonic intravenous fluids were administered in accordance with the standard approach at the time of treatment.

In the present study, we found that severe hyponatremia was related to elevated blood glucose levels, which is a marker of stress. Infections can induce hyperglycemia by increasing the secretion of glucagon, catecholamines, and cortisol. Hyperglycemia also increases serum osmolality, resulting in the efflux of intracellular water and consequently in dilutional hyponatremia (28, 29). We found that increased blood glucose levels (>6.1 mmol/L) and convulsions predicted severe hyponatremia in pediatric bacterial meningitis. We also found that hyponatremia was associated with a shorter duration of symptoms prior to admission, which may have been due to an increased symptom severity resulting in earlier diagnosis. These results imply that the development of hyponatremia has a minimal relationship with time.

This study was a retrospective review of children with bacterial meningitis who received standard care. Repeated serum sodium levels were used to define hyponatremia, and miscellaneous clinical characteristics were analyzed. While this study addresses some interesting clinical questions, it also has several limitations that warrant discussion. Critically, the assessment of these factors and of symptom severity in infants is often inaccurate, a limitation which may have prevented our ability to draw more definitive conclusions regarding causality in the present study. In addition, we have not researched further neurodevelopmental outcomes, such as the intellectual profile, because these data are not yet available. Studies with a larger number of patients may indicate possible sex-related or infecting organism-based differences. Further studies are needed to evaluate the practice variations and effectiveness of treatment of hyponatremia in patients with pediatric bacterial meningitis, as well as the impact of various treatments on patient outcomes. Further studies are also needed to characterize the etiological mechanism and place these findings in the context of other pediatric neurological illnesses.

## Conclusions

Our data suggest that hyponatremia is a common complication in children with bacterial meningitis. Hyponatremia was generally mild and was associated with a longer duration of hospitalization. Moderate hyponatremia was associated with severity of disease; severe hyponatremia was associated with both severity of disease and short-term poor outcome, suggesting that it is an important problem in children with bacterial meningitis. Convulsions and increased blood glucose levels (>6.1 mmol/L) are predictive of severe hyponatremia. As the diagnostic symptoms of hyponatremia during CNS meningitis may be overlooked, we recommend that patients with pediatric bacterial meningitis who exhibit convulsions and increased blood glucose levels should be checked for severe hyponatremia. Further studies are needed to evaluate the effectiveness of treatment of hyponatremia in patients with pediatric bacterial meningitis, as well as the impact of various treatments on patient outcomes. A larger study is required to confirm our findings. Further work is also required to determine the etiological mechanism.

## Ethics Statement

This study was carried out in accordance with the recommendations of Clinical practice guideline on diagnosis and treatment of hyponatremia and The Second Affiliated Hospital & Yuying Children's Hospital of Wenzhou Medical University ethics committee with written informed consent from all subjects. All subjects gave written informed consent in accordance with the Declaration of Helsinki. The protocol was approved by the The Second Affiliated Hospital & Yuying Children's Hospital of Wenzhou Medical University ethics committee.

## Author Contributions

FZ conceptualized and designed the study, designed the data collection sheets, performed all data analyses, drafted the initial manuscript, and approved the final manuscript as submitted. LJ conceptualized the study, participated in drafting the manuscript, and approved the final manuscript as submitted. XY, XS, ZL, and ZY recruited patients for the study, participated in the clinical and imaging examination of all subjects, and approved the final manuscript.

### Conflict of Interest Statement

The authors declare that the research was conducted in the absence of any commercial or financial relationships that could be construed as a potential conflict of interest.
